# Mental Health of Homebound Older Adults in China: The Moderating Effect of Loneliness

**DOI:** 10.1093/geroni/igaa057.313

**Published:** 2020-12-16

**Authors:** Tao Chen, Shuangshuang Wang, Nengliang (Aaron) Yao

**Affiliations:** Shandong University, Jinan, Shandong, China

## Abstract

Homebound older adults are confined to their homes due to physical, mental, or social limitations, which contributes to elevated levels of depression. However, the mental health status of the homebound population in China is relatively overlooked. This study compares mental health status between homebound and non-homebound older adults, and examines the moderation effect of loneliness. The sample consists of 1,301 older adults aged 60 and over (39% homebound, 49% females, mean age = 69) from Shandong Aging and Health Survey, conducted by Shandong Provincial Government in 2019. Mental health status was measured by feelings of depression, not cheerful, bored, not calm or peaceful, and not happy. Compared to non-homebound older adults, homebound older adults tend to be older, lower educated, live in rural areas, and in worse health conditions. Results from generalized linear regression models show that controlling for demographic and physical health status, homebound population were more likely to have worse mental health status than other Chinese older adults. Feeling lonely, isolated, or lack of companionship intensifies the adverse effects of being homebound on older adults’ mental health. Findings from this study suggest that homebound older adults in China had both physical and psychological sufferings. Social programs and interventions may be designed to improve homebound older adults’ mental health. As the number of homebound older adults increases in China, medical care models may be tailored to improve the accessibility of healthcare services among people who are confined to their homes.

